# Transforming text to music using artificial intelligence improves the frontal lobe function of normal older adults

**DOI:** 10.1002/brb3.70007

**Published:** 2024-09-05

**Authors:** Masayuki Satoh, Jun Inoue, Jun‐ichi Ogawa, Ken‐ichi Tabei, Chiaki Kamikawa, Makiko Abe, Ayaka Yoshizawa, Gyo Kitagawa, Yosinori Ota

**Affiliations:** ^1^ Department of Dementia and Neuropsychology, Advanced Institute of Industrial Technology Tokyo Metropolitan Public University Corporation Tokyo Japan; ^2^ Amadeus Code, Co., Ltd Tokyo Japan; ^3^ Department of Music Research Yamaha Music Foundation Tokyo Japan; ^4^ School of Industrial Technology, Advanced Institute of Industrial Technology Tokyo Metropolitan Public University Corporation Tokyo Japan; ^5^ Research Institute of Brain Activation Tokyo Japan

**Keywords:** artificial intelligence, composition, music, Music Trinity Generative Algorithm‐Human Refined, *otokai*

## Abstract

**Introduction:**

Recent advances in artificial intelligence (AI) have been substantial. We investigated the effectiveness of an online meeting in which normal older adults (*otokai*) used a music‐generative AI that transforms text to music (Music Trinity Generative Algorithm‐Human Refined [MusicTGA‐HR]).

**Methods:**

One hundred eighteen community‐dwelling, cognitively normal older adults were recruited through the internet (64 men, 54 women; mean age: 69.4 ± 4.4 years). Using MusicTGA‐HR, the participants chose music that they thought was the most suitable to a given theme. We established 11 classes of 7–10 members and one instructor each. Each class held an online meeting once a week, and each participant presented the music they chose. The other participants and the instructor then commented on the music. Neuropsychological assessments were performed before and after the intervention for 6 months, and the results before and after the intervention were statistically analyzed.

**Results:**

The category and letter word fluencies (WFs) were significantly improved (category WF: *p *= .003; letter WF: *p *= .036), and the time of the Trail‐Making Test‐B was also significantly shortened (*p *= .039). The Brain Assessment, an online cognitive test we developed, showed significant improvement in the memory of numbers (*p *< .001).

**Conclusion:**

The online meeting of the *otokai*, which used music‐generative AI, improved the frontal lobe function and memory of independent normal older adults.

## Graphical abstract

We investigated the effectiveness of an online meeting in which normal older adults (*otokai*) used a music‐generative artificial intelligence (AI) that transforms text to music (Music Trinity Generative Algorithm‐Human Refined [MusicTGA‐HR]). Using MusicTGA‐HR, the participants chose music that they thought was the most suitable to a given theme. Each class held an online meeting once a week for 6 months, and each participant presented the music they selected. As a result, the frontal lobe function and memory were improved.

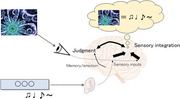

## 1 INTRODUCTION

The number of people with dementia is rising rapidly with the increase in longevity. Approximately 46.8 million people worldwide are estimated to be living with dementia, and 9.9 million new cases of dementia are diagnosed every year (Strøm et al al., 2016). According to Alzheimer's Disease International (Prince et al., 2015), these numbers will nearly double every 20 years to an estimated 74.7 million in 2030 and 131.5 million in 2050, with a large proportion of those individuals living in Asia (Satoh et al., 2020). Recent studies have demonstrated that through adequate intervention, including the control of lifestyle parameters, such as those related to hypertension, physical exercise, or intellectual activities, the occurrence of dementia can be prevented to some degree (Barnes & Yaffe, 2011; Livingston et al., 2017; Satoh et al., 2014/2017/2020; Tabei et al., 2017).

Today in the field of neurology, the effectiveness of the music therapy is established in following neurological diseases or symptoms: dementia (Moreno‐Morales et al., 2020; van der Steen et al., 2018), Parkinson's disease (Zhang et al., 2017; Zhou et al., 2021), stroke (Magee et al., 2017; Van Criekinge et al., 2019), aphasia (García‐Casares et al., 2022; Liu et al., 2022), and unilateral spatial neglect (Long et al., 2023). It is well‐known that music interventions have a significant effect on stress reduction (de Witte et al., 2020/, 2022), and the effectiveness of music therapy was reported about the behavioral and psychological symptoms of dementia (BPSD) (Dyer et al., 2018; Ueda et al., 2013). Later, the effectiveness to cognitive function of older adults with dementia was also reported (Dorris et al., 2021; Ito et al., 2022; Moreno‐Morales et al., 2020). Recently, the relationships between kinds of music activities and the effects to health and well‐being have been reported (Dingle et al., 2021). They showed the effectiveness of receptive and intentional music listening to main reduction, shared music listening to the enhancement of social connections in older adults, music listening and carer singing to agitation of people with dementia, group singing, playing a musical instrument, and dance and movement with music programs to the improvement of cognitive health and well‐being, and rapping, songwriting, and composition to the well‐being of marginalized people. Musical activities, such as playing instruments and music composition, require long‐term specialized training that generally begins in childhood. It is quite challenging for normal older individuals to start to compose musical pieces if they have not had prior musical training. Therefore, these individuals are more likely to participate in musical activities such as listening to music and singing songs. Recently, the development of artificial intelligence (AI) has led to substantial advancements in many fields. For example, Chat Generative Pre‐trained Transformer (ChatGPT) allows a user to input words or text and generate appropriate responses to inquiries or questions. In image generation AI, such as Stable Diffusion Online (https://stablediffusionai.org/#home) or Bing Image Creator (https://www.bing.com/create), novel images can be created using input text. The musical version of such the generative AI is Music Trinity Generative Algorithm‐Human Refined (MusicTGA‐HR; https://www.amadeuscode.com/musictga‐hr). MusicTGA‐HR has decomposed data for musical components, including melody, rhythm, harmony, timbre, instruments, instrumentation, and style. By combining these features and relating them to corresponding input text, we can obtain an almost unlimited selection of high‐preference music. Making music, namely, composition, requires in‐depth learning and training which often starts during childhood. However, MusicTGA‐HR enables musically naïve persons to choose the most suitable music for their image or concept. Many YouTubers use MusicTGA‐HR as background music for their videos, and more than 44,000 musical pieces are generated each month worldwide using this platform.

Various mental activities, including drawing, ceramic art, dressmaking, and cooking, are used for the cognitive stimulation training, a type of nonpharmacological intervention aimed at improving the quality of life of older individuals (Societas Neurologica Japonica, 2017). In Japan, another type of cognitive stimulation training involves the *haiku*, the shortest fixed form poem in the world. In meetings called *kukai*, participants write *haikus* for a given theme and present it in front of the other members of the meeting. Then, the instructor makes some comments on each *haiku*. Originally, the *kukai* was held in‐person; however, after the outbreak of coronavirus disease‐2019 (COVID‐19), in‐person meetings became difficult. Therefore, online *kukais* are now often held using videoconference systems. Based on this background, we hypothesized that MusicTGA‐HR may have applications as a nonpharmacological therapy for improvement of the cognitive function of older adults. Using MusicTGA‐HR, individuals can choose music that they think is most suitable for a given theme. At online meetings, each participant presents the music they chose, and the other participants state their impressions of that music. The instructor will also comment on the music. According to the term *kukai*, which is used for meetings to discuss *haiku*, as mentioned above, we named this type of musical meeting *otokai*. In Japanese, “oto” and “kai” mean music/sound and meeting, respectively; therefore, *otokai* means a meeting of music/sound.

The aim of this study was to investigate the effectiveness of the *otokai* for improving the cognitive functions of community‐dwelling, normal older individuals. Using a videoconference system, we held the *otokai* for 6 months and carried out cognitive assessments before and after the intervention period. The primary outcome was changes in neuropsychological batteries before and after the intervention period. We expect that our results may have applications in dementia prevention, which is currently one of the most important and pressing problems in the world.

## 2 MATERIALS AND METHODS

### 2.1 Subjects

**FIGURE 1:**
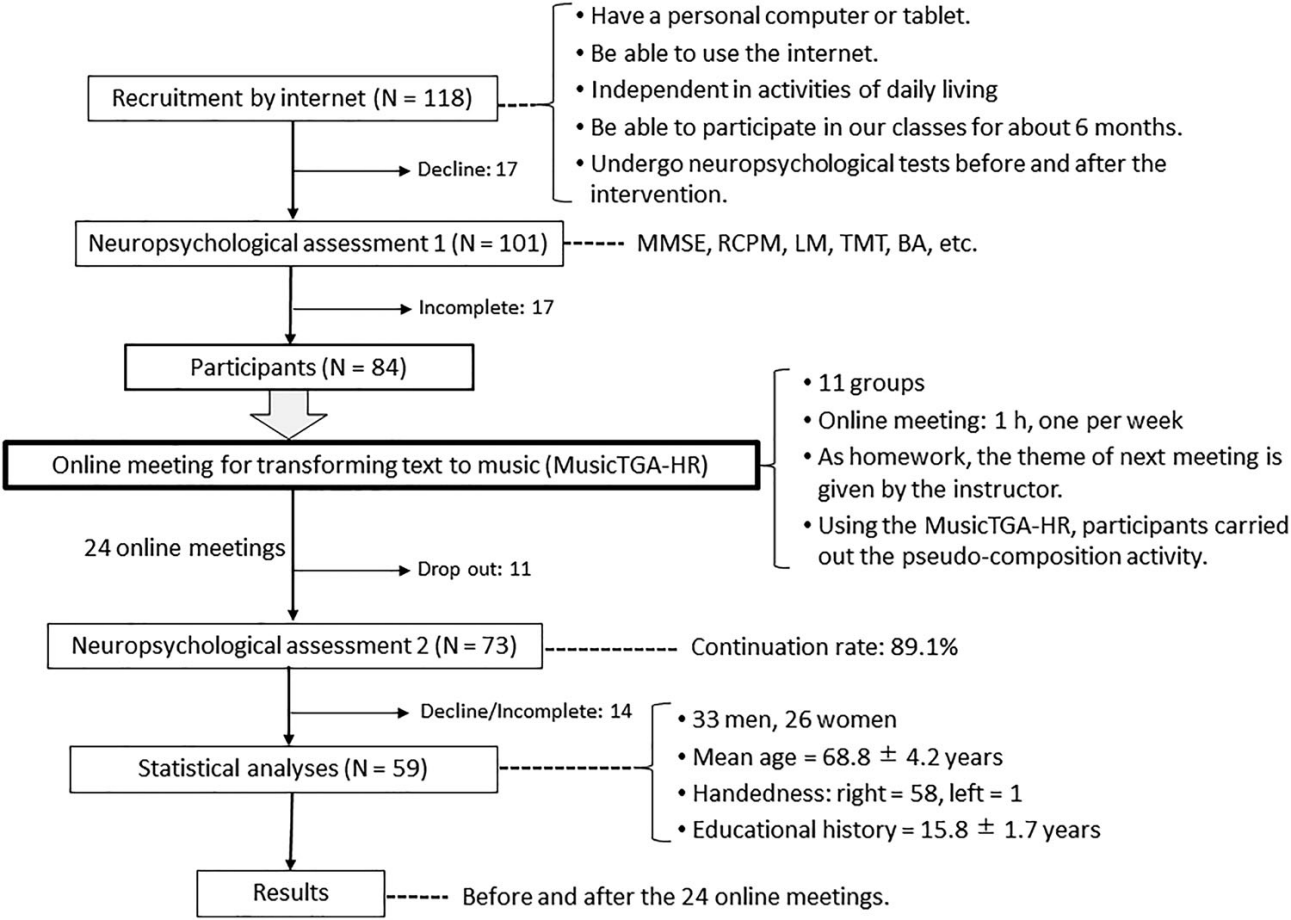
Diagram of the flow of this study. BA, Brain Assessment; LM, logical memory; MMSE, Mini‐Mental State Examination; MusicTGA‐HR, Music Trinity Generative Algorithm‐Human Refined; *N*, number; RCPM, Raven's Colored Progressive Matrices; TMT, Trail Making Test.

The number of subjects was decided based on our previous study (Tabei et al., 2023). In this study, we investigated the effectiveness of the online physical exercise with music accompaniment for older adults. One hundred and fourteen subjects participated in the online exercise class for 6 months, and before and after the intervention period, neuropsychological examinations were performed. Finally, the results of 75 subjects were used for statistical analysis, and we found the significant improvement on the N‐back task which belonged to a frontal lobe function. So, in the present study, we planned to establish 10 groups of 12 participants each, for a total of 120 participants (Figure 1). We recruited participants through the internet. Because our intervention was carried out through the internet, we expected that the recruited participants would have the skill for digital devices (Satoh et al., 2023). We announced this study by sending direct emails to approximately one million adults who had a SAISON credit card, issued by the parent company of the Research Institute of Brain Activation. The inclusion criteria for participants were as follows: (a) over 65 years old, (b) psychologically healthy, (c) good eyesight, (d) able to hear instructions clearly, (e) able to function independently in most aspects of life, (f) have a personal computer or tablet, (g) able to use digital devices, (h) able to participate in our classes for approximately 6 months, and (i) agreed with the performance of a neuropsychological examination before and after the intervention period (Satoh et al., 2023). Applicants were excluded if they met any of the following exclusion criteria: (a) apparent history of cerebrovascular attack; (b) presence of chronic exhausting disease, such as malignancy or infection; (c) presence of severe cardiac, respiratory, and/or renal disabilities; (d) use of drugs that might adversely affect cognition (antidepressants and antipsychotics); (e) previous diagnosis of dementia; or (f) unable to use digital devices (Satoh et al., 2023). One hundred eighteen participants were recruited on a first‐come basis (64 men, 54 women; mean age: 69.4 ± 4.4 years; Figure 1).

### 2.2 Online meeting for transforming text to music using AI (*otokai*)

The instructors were professional musicians who also held private licenses as musical trainers with the YAMAHA Music Foundation. The author JO is the head and the mentor of YAMAHA's musical trainers, and he selected the *otokai* trainer who is, he thought, adequate for this study, based on their achievement and suitability. Prior to the beginning of the study, the exercise of the *otokai* instructors was performed online, dividing them into two groups. First, the author MS lectured the aim and the background of this study. Then, the methods of the *otokai* were explained by the author MS and JO as mentioned below. Lastly, the author KT and AY showed how to operate the MusicTGA‐HR. Namely, we taught the instructors about the purpose and contents of the study, the way of holding *otokais*, the positive feedback of comments to each subject, as well as the operating procedures of MusicTGA‐HR. Total time of the exercise was almost 2 h. The instructor can question to the authors at any time after the exercise. After excluding individuals who declined to participate, we established 11 classes of 7−10 members and one instructor each. Each participant got the operation manual of MusicTGA‐HR online, and, almost a week before starting the intervention, the participants of each group were explained online about the aim of this study, procedure of online meeting, and how to operate MusicTGA‐HR by the author JO, AY, and the instructor of the group. Each meeting was held as follows. First, the theme of the next online meeting was set and communicated via email to each member. Using MusicTGA‐HR, the participant then chose the music they thought was most suitable to given the theme. The file of the music could be recorded in the PC/tablet of each participant. Second, in each online meeting, each member presented their music and explained the reason why they chose it. The instructor commented on the music and encouraged other members to also comment on the music. Third, the instructor presented the music they chose and recorded in their PC in advance in the same way as the members. Finally, the instructor described the theme for the next meeting, and the meeting ended. Based on the term *kukai*, we named this type of meeting *otokai*. For the online meeting, a videoconference system (Zoom, Zoom Video Communications, Inc.) was used. Online meetings of *otokai* were held once a week and were 1 h long. In total, 24 online meetings were held over the course of 6 months.

The theme gradually became more difficult, moving from concrete to abstract (Table 1). The themes for the last four meetings (from the 21st to 24th meeting) were autobiographical materials; the participants chose their own pictures or movies, which showed things they were proud of (e.g., a journey abroad, prizes their children had won, and their hobbies).

**TABLE 1:** The theme of each meeting of *otokai*.

Section	Meeting No.	Theme
**1. Concrete pictures**	1	Mt. Fuji
	2	A train
	3	A dinosaur
	4	Carps in a pond
**2. Scenes of human activities**	5	Fireworks
	6	Festivals
	7	Playing a piano
	8	Running in a marathon
**3. Famous sentences**	9	A poem by Issa Kobayashi
	10	A proverb
	11	A saying by a famous historical person
	12	A poem by Shuntaro Tanigawa
**4. Concrete short movies**	13	A sleeping kitten
	14	Seashore with large waves
	15	A toy showing the turning of a Ferris wheel
	16	A walking tiger
**5. Abstract pictures**	17	A figure of a Mandelbrot set
	18	A painting by Wassily Kandinsky
	19	A painting by Wassily Kandinsky
	20	A movie with shining lights
**6. Autobiographical materials**	21	Pictures and movies chosen by participants
	22	
	23	
	24	

### 2.3 Neuropsychological assessments

Neuropsychological assessments were performed online within 2 weeks before starting and after ending the intervention. Each assessment required almost 1 h and was carried out at one occasion. Subjects were not paid. For neuropsychological assessments, the tests were nearly identical to those used in the Mihama‐Kiho Project, which investigated the effects of physical exercise with music accompaniment in normal and cognitively impaired older adults (Satoh et al., 2014/2017/2020/2023; Tabei et al., 2017/2023). To quantify intellectual function, the Mini‐Mental State Examination (MMSE) (Folstein et al., 1975) and the Japanese version of the Raven's Colored Progressive Matrices (RCPM) (Raven, 1995) were administered. We were going to exclude the subject who revealed the abnormal score of the MMSE, but all participants showed normal scores (24–30). RCPM not only provides a score but also measures the performance time, which reflects the psychomotor speed of the participant. Memory was evaluated using logical memory I and II of the Rivermead Behavioral Memory Test (Wilson et al., 1985), which consists of immediate and delayed recall of a short story. Assessment of constructional ability was based on the method described by Strub and Black (2000). A cube was shown to the examinees, and they were asked to draw it. Their drawing was scored by assigning one of four possible grades (0: poor, 1: fair, 2: good, and 3: excellent). The Mie Constructional Apraxia Scale (MCAS) was also used to assess constructional visuospatial ability (Satoh et al., 2016). The MCAS is designed to assess constructional disabilities by evaluating not only the shape of a drawn Necker‐cube but also the drawing process. Higher scores are indicative of worse symptoms. Additional details are available in our previous paper (Satoh et al., 2016). Frontal function was assessed by two types of tasks: word fluency (WF) and Trail‐Making Test A and B (TMT‐A/B). The WF test consisted of two domains: category and letters. For the categorical WF, participants were asked to name as many animals as possible in 1 min. For the letter WF, for each of four phonemes (*ka*, *sa*, *ta*, and *te*), the participants were asked to name objects that have that phoneme at the beginning of the word (Dohi et al., 1992). We used the average scores of these four phonemes for statistical analyses. It is generally accepted that the cognitive processing of categorical and letter WFs is somewhat different; categorical WF is more reflective of memory function than letter WF (Satoh et al., 2017). These neuropsychological assessments were administered before and after the intervention for 6 months.

We also used an online cognitive test that we recently developed, named Brain Assessment (BA) (Satoh et al., 2021a, 2021b, 2022). The BA covers five fields: number memory, word memory, mental rotation, working memory (N‐back test), and judgment task. The cardinal features of the BA include five different versions to avoid habituation, conciseness (30 min), an automated scoring system, easy access on a website, and basic data based on a large population of 5000 participants with a wide age range of 40−89 years. More details are given in our previous papers (Satoh et al., 2021a, 2021b, 2022).

### 2.4 Statistical analyses

Statistical analyses were performed based on selection of appropriate tests from a statistics textbook (Tsushima, 2007). For neuropsychological tests, including the BA, statistical analyses were performed as follows: changes before and after the 6‐month intervention period were analyzed. The Shapiro–Wilk test was used to evaluate normality. If the result was parametric, a paired *t* test was used; otherwise the Wilcoxon signed–rank test was used. We regarded the result as significant if the *p* value was less than0.05. We also calculated the effect sizes. All statistical analyses were performed using IBM SPSS Statistics 27 software.

## 3 RESULTS

**FIGURE 2:**
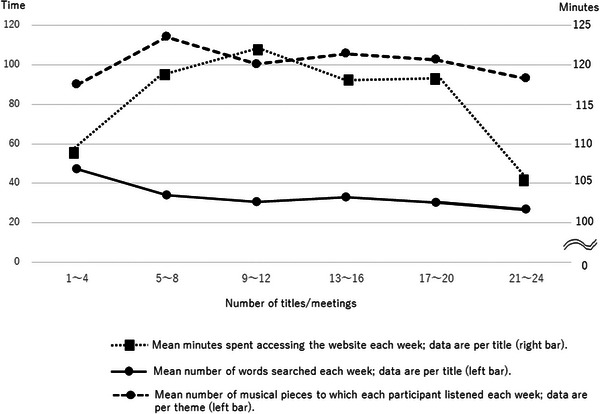
The mean minutes spent accessing the website, mean numbers of words searched on their PCs/tablets, and mean number of musical pieces to which each participant listened each week.

During the 6‐month intervention period, 11 individuals dropped out of the study due to schedule conflicts, the meetings being different from their expectations, health problems, or needing to provide nursing care for their spouses. The continuation rate for 6 months was 89.1%. Figure 2 shows the mean number of minutes participants spent accessing the website, the mean number of words searched on their PCs/tablets, and the number of musical pieces to which each individual listened within a week. These numbers were automatically recorded by the MusicTGA‐HR system. For each theme, individuals spent approximately 100−120 min on the intervention each week. They searched for approximately 40 words and listened to 100 musical pieces each week. Although the theme became more difficult over time, the number of words searched and the number of musical pieces the participants listened to remained almost constant (from meetings 5−20). However, for the last four meetings (meetings 21−24), the time spent accessing the website decreased. These changes suggested that the participants already had a specific image in mind when they selected their autobiographical materials.

The total number of participants who were eligible to take neuropsychological examinations after the intervention was 73 (Figure 1). Because 14 participants declined or did not fully complete the examination, the results of 59 participants (33 men, 26 women; mean age: 68.8 ± 4.2 years; education history: 15.8 ± 1.7 years) were analyzed.

The results of neuropsychological examinations are shown in Table 2. The category and letter WFs were significantly improved after the 6‐month intervention (category WF: *p *= .003; letter WF: *p *= .036; Table 2). The time of the TMT‐B was also significantly shortened (*p *= .039). As for the BA, a significant improvement was observed in terms of the memory of numbers (*p *< .001; Table 2). From these results, we concluded that the intervention with the online meeting using MusicTGA‐HR to convert text to music improved frontal lobe function and memory.

**TABLE 2:** Results of neuropsychological batteries and Brain Assessment (BA) following the intervention using the Music Trinity Generative Algorithm‐Human Refined (MusicTGA‐HR) for 6 months.

		Before	After	Effect size	*p* Value
**Intellect**	**MMSE**	29.0 ± 1.0	29.0 ± 1.0	0	.49
	**RCPM**				
	Score	32.3 ± 2.4	32.7 ± 2.2	0.17	.39
	Time	234 ± 41	230 ± 53	0.08	.097
**Memory**	**LM‐I**	13.5 ± 3.3	13.8 ± 3.5	0.09	.44
	**LM‐II**	12.7 ± 3.3	13.0 ± 3.4	0.09	.39
**Visuospatial**	**Necker**				
	Score	2.9 ± 0.2	3.0 ± 0.1	0.58	.32
	Time	20.6 ± 8.3	20.9 ± 9.5	0.03	.64
	**Construction**	17.6 ± 0.6	17.6 ± 0.6	0.33	.74
**Frontal function**	**WF**				
	Category	16.0 ± 3.8	17.8 ± 4.3	0.44	**.003**
	Letters	14.0 ± 2.9	14.8 ± 2.5	0.29	**.036**
	**TMT‐A**	94 ± 46	97 ± 55	0.06	.96
	**TMT‐B**	110 ± 52	98 ± 54	0.23	**.039**
***Brain Assessment (BA)***					
**Memory**	Numbers	55.6 ± 14.0	60.0 ± 14.8	0.31	**<.001**
	Words	52.9 ± 9.9	54.0 ± 9.8	0.11	.13
**Visuospatial**	MRT	52.1 ± 12.1	51.6 ± 13.6	0.04	.58
**Working memory**	N‐back test	58.1 ± 14.4	58.6 ± 16.2	0.03	.57
**General intellect**	Judgment	53.8 ± 15.6	54.8 ± 16.3	0.06	.30
**Total score**		54.4 ± 10.0	55.5 ± 10.4	0.11	.082

*Note*: Bold letters indicate statistical significance.

LM, logical memory; MMSE, Mini‐Mental State Examination; RCPM, Raven's Colored Progressive Matrices; TMT, Trail Making Test; WF, word fluency.

## 4 DISCUSSION

In this study, we carried out an intervention in cognitively normal older adults via online meetings using AI that transformed text to music (MusicTGA‐HR). This activity was named *otokai*, meaning the meeting of music/sound. According to a preset theme, participants chose music made by the AI, selecting musical pieces that were most suitable to their image and preferences. Professional musicians acted as instructors and provided positive feedback on the music. The meetings were held once a week for 6 months and were 1 h long each. The continuation rate was 89.1%. Neuropsychological assessments revealed that frontal lobe function and memory were significantly improved after 6 months.

Music perception involves complex brain functions underlying acoustic analysis, auditory memory, auditory scene analysis, and processing of musical syntax and semantics, and potentially affects emotion, influences the automatic nervous system, the hormonal and immune systems, and activates (pre)motor representations (Koelsch & Siebel, 2005). Many brain regions participate in the music perception: neocortical regions, insula, cingulate cortex, primary and secondary somatosensory cortex, premotor cortex, frontal operculum, and auditory cortex (Koelsch et al., 2021). It was suggested that music training‐related pathway plasticity facilitated the right hemisphere ventral stream information transfer that connects the middle temporal lobe with the inferior frontal cortex via the extreme capsule, supporting an improved sound‐to‐meaning mapping in music (Oechslin et al., 2018). Brain network connectivity can change after receptive music‐based intervention in cognitively unimpaired older adults: comparing pre‐ and post‐intervention showed significant increase in functional connectivity between auditory regions and medial prefrontal cortex (Quinci et al., 2022). We can say that the frontal lobes participate in the formation of music perception.

Notably, in the *otokai* in this study, the proportion of male participants was higher than that of other in‐person nonpharmacological interventions. For example, for the current study, 55.9% of participants were men, whereas in an in‐person intervention with physical exercise combined with music accompaniment previously reported by our group (the Mihama‐Kiho project), the percentage of male participants was only 20% (Satoh et al., 2014). Due to the COVID‐19 outbreak, we carried out the same intervention using a videoconference system (Satoh et al., 2023; Tabei et al., 2023), and the percentage increased to approximately 50% (Satoh et al., 2023). Interestingly, a questionnaire administered to study participants showed that more than half would not have participated in the physical exercise plus music accompaniment class if it had been held in‐person. Their main reasons were the risk of COVID‐19 infection, trouble with getting to the exercise site, and discomfort in interactions with others (Satoh et al., 2023). In many regions in Japan, older men tend to have decreased social activity, and this problem has yet to be solved (Morinaga et al., 2018). However, the online *otokai* may facilitate participation by older men in nonpharmacological interventions, increasing the percentage of male participants compared with in‐person classes.

The *otokai* also showed a relatively high continuation rate (89.1% over the 6‐month intervention). To the best of our knowledge, the continuation rate for nonpharmacological interventions in older adults ranges from 65% to 100% for interventions lasting 3−6 months (Gajewski & Falkenstein, 2012; Kraus‐Sorio et al., 2022; Suzuki et al., 2014). Therefore, the continuation rate obtained in this study is sufficient and consistent with those of similar studies. Our novel approach using AI for music composition and the gradual increase in difficulty may have helped keep participants engaged.

**FIGURE 3:**
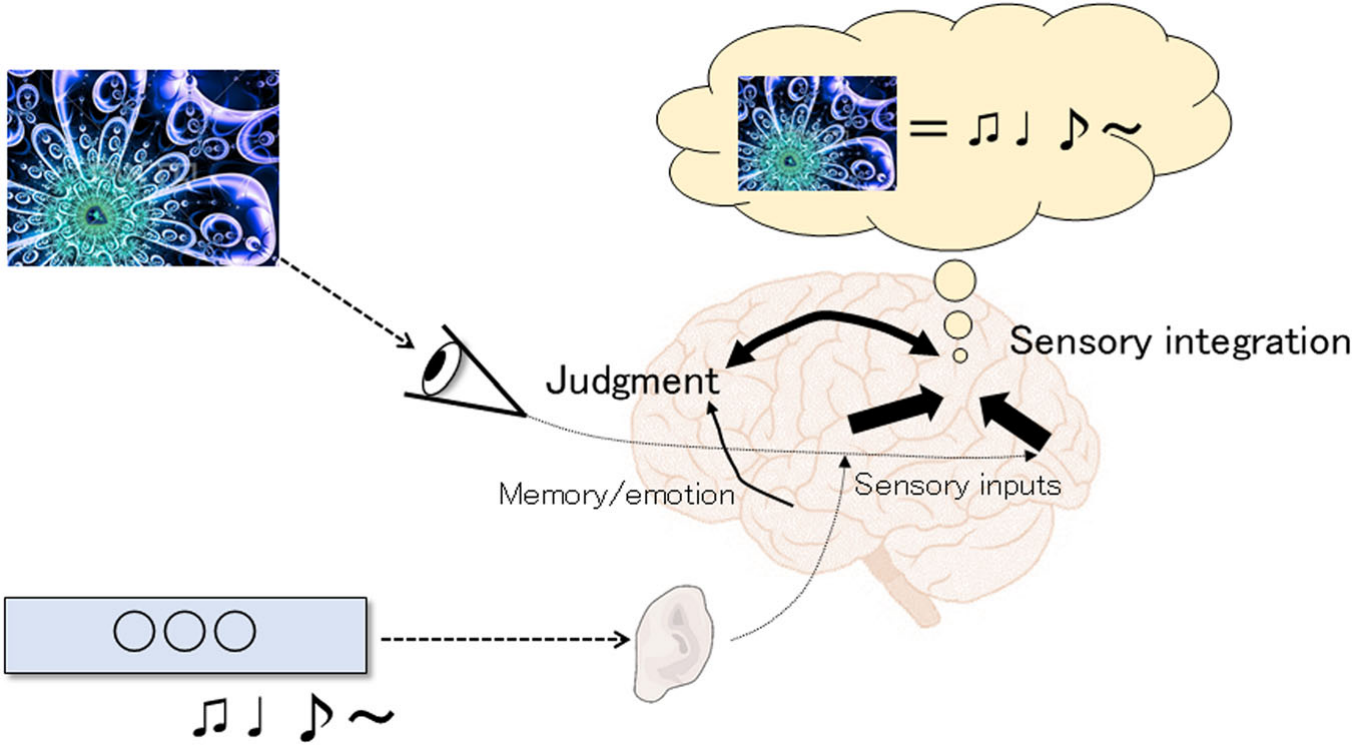
The cognitive processing in the brain that might occur during the intervention used in the current study. The figure shows the so‐called Mandelbrot set, which was used at the 17th *otokai*.

The most important characteristic of the *otokai* was the significant improvements observed in frontal lobe function and memory after the 6‐month intervention. As shown in Figure 3, the participant viewed the figure, which was the Mandelbrot set used as the theme for the 17th meeting in this study. This visual stimulus was perceived at the occipital lobe, and then, when the participant listened to music using MusicTGA‐HR, the information was processed via the temporal lobes. The inferior portion of the parietal lobe, particularly the angular gyrus, is the integration site for multiple sensory information. The information for the figure and music may be integrated, and the frontal lobe then functions to judge whether the two pieces of sensory information conform to each other. The participants compared the music composed by MusicTGA‐HR and the given theme almost one hundred times a week. This process might stimulate their cognitive function. During online meetings, the instructor would ask each participant to describe personal experiences related to the theme. In cases in which a familiar object, such as Mt. Fuji, was the visual stimulus, episodic and semantic memory, involving emotions related to personal experiences, may be evoked. By repeating these processes for 6 months, we hoped to improve frontal lobe function and memory.

We also compared the results of neuropsychological assessments between male and female subjects. The age and education level were almost the same between them (age, *p *= .90; education level, *p *= .68) (Table A1). As for the neuropsychological tests, the changes of the scores of the MMSE and RCPM were significantly better in female compared to male subjects (MMSE, *p *= .007; RCPM, *p *= .029) (Table A2). After the intervention, the scores slightly worsen in male (*p *= .050), but those of female subjects slightly improved (*p *= .16). As long as we know, there is no report which showed significant gender differences in nonpharmacological interventions to dementia. The present results might suggest that the effects of nonpharmacological interventions are different depending on the gender. It is a very interesting problem, and remains to be investigated in the future.

The current study had several limitations. First, the number of participants who were included in the statistical analyses was not so large (*n* = 59). Thus, additional studies with more participants may be needed in order to confirm the results. Second, the intervention period was 6 months. Longer interventions may give different results and the data of sustainability of cognitive improvements. Third, the instructors were professional musicians, and therefore, it may be necessary to train more musicians in order to expand the *otokai* to other locations worldwide. Fourth, there was no control group in the present study. It is possible that the conversational nature of the *otokai* may have improved participants’ verbal fluency. We are now planning to carry out the comparison of cognitive changes between *otokai* and another nonpharmacological intervention group in order to strengthen the causality of the findings. Lastly, the participants in the *otokai* need information technology literacy. We expect that the use of the internet does not represent a barrier for today's older population to participate in the *otokai* (Satoh et al., 2023). According to the Annual Report on the Ageing Society 2021 published by the Cabinet Office in Japan (Annual Report on the Ageing Society, 2021), approximately 74% and 58% of septuagenarians and octogenarians, respectively, utilize the internet, and the rates have increased almost two‐ to three‐fold compared with the results from 2010 (septuagenarians: 39.2%, octogenarians: 20.3%) (Annual Report on the Ageing Society, 2010). The Communication Usage Trend Survey (2020) performed by the Japanese Ministry of Internal Affairs and Communications showed that 53.9% of people over 65 years old use the internet (men: 64.4%, women: 45.7%). Because utilization of the internet by older adults is increasing yearly, we expect that more older adults will be able to participate in the *otokai* more easily in the future. In order to introduce the tech‐savvy individuals to *otokai*, in‐person activity will be needed by conquering the problem of shortage of the number of instructors.

In the *otokai*, men accounted for more than half of all participants. This supports the observation that men tend to be more willing to participate in nonpharmacological activities if they are held online, not in‐person. Moreover, the *otokai* can be held in place of in‐person activities despite restrictions due to health concerns, such as the outbreak of COVID‐19, and can also be used for inhabitants of remote and islands areas. Therefore, this approach may be useful in countries in which the aging population is growing.

## AUTHOR CONTRIBUTIONS

**Masayuki Satoh**: Conceptualization; methodology; investigation; formal analysis; supervision; project administration; visualization; writing—original draft; writing—review and editing; resources; data curation. **Jun Inoue**: Conceptualization; methodology; investigation; supervision; project administration; resources. **Jun‐ichi Ogawa**: Conceptualization; methodology; investigation; project administration; resources. **Ken‐ichi Tabei**: Conceptualization; methodology; investigation; project administration; resources; data curation. **Chiaki Kamikawa**: Methodology; data curation; investigation; project administration. **Makiko Abe**: Conceptualization; methodology; data curation; investigation; project administration; resources. **Ayaka Yoshizawa**: Methodology; data curation; investigation; project administration. **Gyo Kitagawa**: Conceptualization; methodology; investigation; project administration; resources. **Yosinori Ota**: Conceptualization; methodology; investigation; project administration; resources.

## FUNDING

The Department of Dementia and Neuropsychology, Advanced Institute of Industrial Technology, Tokyo Metropolitan Public University Corporation was established using donations provided by the Research Institute of Brain Activation.

## CONFLICT OF INTEREST STATEMENT

The authors declare no conflict of interest.

### PEER REVIEW

The peer review history for this article is available at https://publons.com/publon/10.1002/brb3.70007

## Data Availability

All data analyzed during this study are included in this article. Further enquiries can be directed to the corresponding author.

## 1

**TABLE A1:** Comparison of characteristics between male and female subjects.

		Male	Female	*p* Value
**Age**	Mean	69.0	69.0	.90
	s.d.	4.5	3.9	
**Edu**.	Mean	15.8	15.6	.68
	s.d.	1.3	2.2	

Edu., education; s.d., standard deviation.

**TABLE A2:** Comparison of the results of neuropsychological tests between male and female subjects.

Tests		Male			Female		*p* Value
	Before	After	Dif	Before	After	Dif	(Dif: male vs. female)
**MMSE**							
Mean	29.2	28.7	−0.5	28.9	29.3	0.4	**.007**
s.d.	1.1	1.1		1.3	1.0		
*p*‐value (before vs. after)	.050			.16			
**RCPM_score**							
Mean	32.6	32.3	−0.3	32.3	33.2	0.9	**.029**
s.d.	2.7	2.4		2.1	1.9		
*p*‐value (before vs. after)	.63			.11			
**RCPM_time**							
Mean	230	217	−13	238	246	8	.11
s.d.	45	40		37	63		
*p*‐value (before vs. after)	.23			.61			
**LM‐I**							
Mean	12.5	13.5	1	14.6	14.2	−0.4	.095
s.d.	10.2	11.8		3.1	3.7		
*p*‐value (before vs. after)	.26			.65			
**LM‐II**							
Mean	12.0	12.7	0.7	13.5	13.4	−0.1	.24
s.d.	3.3	3.8		3.3	2.9		
*p*‐value (before vs. after)	.43			.86			
**Necker_score**							
Mean	2.9	3.0	0.1	3.0	3.0	0	.36
s.d.	0.25	0		0.19	0.19		
*p*‐value (before vs. after)	.16			1.0			
**Necker_time**							
Mean	18.2	19.0	0.8	23.4	23.1	−0.3	.64
s.d.	6.3	8.3		9.6	10.4		
*p*‐value (before vs. after)	.66			.91			
**Construction**							
Mean	17.6	17.6	0	17.7	17.6	−0.1	.48
s.d.	0.6	0.6		0.6	0.6		
*p*‐value (before vs. after)	.84			.47			
**WF_category**							
Mean	15.5	17.7	2.2	16.6	18.0	1.4	.47
s.d.	3.4	3.4		4.2	5.2		
*p*‐value (before vs. after)	.012			.29			
**WF_letters**							
Mean	10.5	10.8	0.3	11.1	11.3	0.2	.87
s.d.	2.6	3.0		3.9	3.6		
*p*‐value (before vs. after)	.71			.89			
**TMT‐A**							
Mean	96.5	95.8	−0.7	90.4	98.1	7.7	.53
s.d.	42.7	50.9		51.2	59.4		
*p*‐value (before vs. after)	.95			.62			
**TMT‐B**							
Mean	116	108	−8	104	86	−18	.57
s.d.	45	55		60	51		
*p*‐value (before vs. after)	.52			.25			

Note: Bold letters indicate statistical significance.Dif, difference; MMSE, Mini‐Mental State Examination, RCPM, Raven's Colored Progressive Matrices; s.d., standard deviation; TMT, Trail‐Making Test; vs., versus; WF, word fluency.
